# Projected burden and distribution of elevated blood pressure levels and its consequence among adolescents in sub-Saharan Africa

**DOI:** 10.7189/jogh.14.04136

**Published:** 2024-06-28

**Authors:** Alexander Chen, Ana O Mocumbi, Dike B Ojji, Laura Waite, Yih-Kai Chan, Justin Beilby, David S Celermajer, Benedicta Ngwenchi Nkeh-Chungag, Albertino Damasceno, Jim Codde, Simon Stewart

**Affiliations:** 1 Torrens University Australia, Adelaide, South Australia, Australia; 2 Universidade Eduardo Mondlane, Maputo, Mozambique; 3 Instituto Nacional de Saúde, Marracuene, Mozambique; 4 University of Abuja, Abuja, Nigeria; 5 University of Cape Town, Cape Town, South Africa; 6 South Eastern Melbourne Primary Health Network Australia, Victorian Department of Health, Melbourne, Victoria, Australia; 7 Australian Catholic University, Melbourne, Victoria, Australia; 8 University of Sydney, Sydney, New South Wales, Australia; 9 Walter Sisulu University, Mthatha, South Africa; 10 Institute for Health Research, University of Notre Dame Australia, Fremantle, Western Australia, Australia

## Abstract

**Background:**

There is minimal data on the number of adolescents in sub-Saharan Africa (SSA) with elevated blood pressure (BP) at increased risk of future cardiovascular events. Combining country-specific population data with data derived from two previously conducted meta-analyses (one African-specific, one based on international cohorts), we sought to address this knowledge deficit.

**Methods:**

We used meta-analysis data from 37 926 adolescents participating in 36 contemporary SSA studies to generate sex-specific proportions of adolescents aged 10–14 and 15–19 years with elevated BP. The estimates were applied to the 2021 World Bank population data for each country in SSA. We then applied the rate of cardiovascular events attributable to elevated BP levels, derived from a meta-analysis of 17 observational, longitudinal cohort studies comprising 4.5 million young adults (non-African), to determine the excess number of cardiovascular events linked to hypertension among those aged 15–19 years transitioning to adulthood.

**Results:**

The estimated prevalence of elevated BP among male and female adolescents aged 10–14 years living in SSA was 7.2% (95% confidence interval (CI) = 4.9–9.9) and 6.9% (95% CI = 4.7–9.5), respectively, which increased to 13.0% (95% CI = 10.6–15.6) and 12.5% (95% CI = 10.4–15.3) among male and female adolescents aged 15–19 years, respectively. Consequently, we estimate that 13.6/138.0 million (95% CI = 10.4–17.3) male and 12.9/135.7 million (95% CI = 9.83–16.3) female adolescents living in SSA have elevated BP. Among the estimated 16.1 million adolescents aged 15–19 years with elevated BP approaching adulthood, the projected excess in cardiovascular events attributable to hypertension (vs normotension) is 201 000 (95% CI = 115 000–322 000) to 503 000 (95% CI = 286 000–805 000) over the next 10–25 years.

**Conclusions:**

Based on the best available data, we estimate that 26.5 million adolescents living in SSA have elevated BP. If left undetected and untreated among those approaching adulthood (those aged 15–19 years), they will experience >0.5 million excess cardiovascular events associated with persistently elevated BP within the next 25 years.

**Registration:**

PROSPERO: CRD42022297948.

Elevated blood pressure (BP), otherwise known as hypertension when found in adults, is a highly preventable condition affecting 1.2 billion people worldwide [1]. When left undetected and untreated, it results in end-organ damage linked to potentially fatal conditions such as hypertensive heart failure and stroke [2]. In high-income countries, these conditions typically affect individuals older than 60 years [3]. However the consequences of undetected/untreated hypertension are evident in much younger individuals living in low-income/low-resource countries, reflecting greater socioeconomic vulnerability and a paucity of preventative health services [4]. This accelerated pattern of hypertensive-related conditions often results in high-levels of morbidity and premature mortality. This phenomenon is particularly evident in the populous countries of sub-Saharan Africa (SSA), where any negative impact on life-expectancy is particularly devastating from an economic productivity/prosperity perspective [5]. Historically, the high rate of hypertension observed in SSA can be attributed to diseases related to poverty and malnutrition, particularly during times of armed conflict [6,7]. However, with rapid economic development and urbanisation, future hypertension and its often fatal consequences will likely reflect an increase in risk factors, such as rising obesity levels and increased salt intake among children and adolescents [8]. Based on the available evidence (with many more representative surveillance studies needed), a recent meta-analysis found a pooled prevalence of 30% (95% confidence interval (CI) = 27–34) for hypertension among adults living in SSA [9], although there was a wide dispersion of estimates according to the location and age of studied populations. Similarly, as part of a unique meta-analysis of available data, we recently reported that around one in 10 males and females aged 10–19 years (adolescents) living in SSA have elevated BP [10].

To prevent the next generation of adolescents in SSA at high risk of entering adulthood with hypertension and then prematurely dying from hypertension-related conditions at a young age, there is an urgent need for more proactive BP screening and treatment programmes. Before any such action is taken or funded, we must calculate how many adolescents in each country/region of SSA have elevated BP and how many will suffer a cardiovascular event in adulthood if they remain untreated/undetected [10]. We are unaware of any previous reports on this critical information.

Based on this, we aimed to combine the best available data on the prevalence of elevated BP among adolescents living in SSA with published/known population data for the region to generate robust estimates of the total number and distribution of male and female adolescents aged 10–14 and 15–19 years with elevated BP in the region. We then aimed to use these estimates to project the number of excess cardiovascular events associated with persistently elevated BP among those aged 15–19 years as they enter early adulthood.

## METHODS

**Figure 1:**
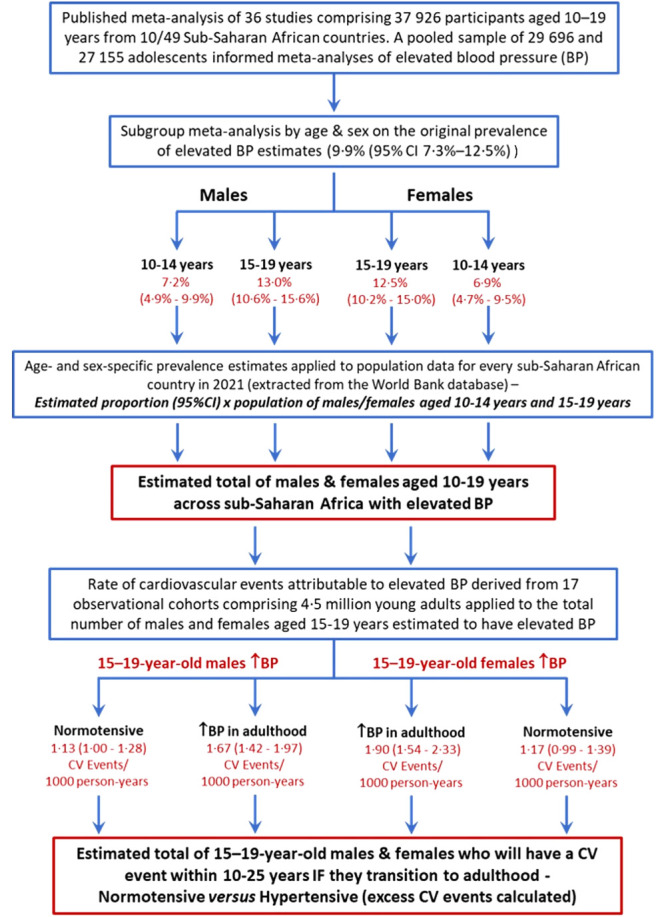
The study schema according to the GATHER guidelines [11], identifying the sources of data and the method of application. It shows how we first refined pre-existing meta-analysis data (levels 1 and 2) to derive more granular age- and sex-specific prevalence estimates of elevated BP among adolescents living in SSA (level 3) that we then applied to population data for each SSA country (level 4) to derive total numbers of affected adolescents in SSA (level 4). Finally, we applied meta-analysis findings on the consequence of elevated BP in young adults (level 5) to adolescent males and females aged 15–19 years to project the total number of cardiovascular events linked to normotension vs hypertension (level 6) to estimate the excess number of events that will occur in the population cohort if nothing is done to reduce their BP levels in adulthood.

As detailed below, along with country-by-country population data for the region, we used data from two previously published meta-analyses that provided the best available evidence on the underlying prevalence of elevated BP among adolescents living in SSA [10], as well as the rate of future cardiovascular events among young adults according to increasingly elevated BP levels/hypertensive status [11]. **Figure 1** summarises how we combined these data to generate the population estimates and future cardiovascular events within a subset of cases. Accordingly, this study (including our methods and data reporting) conforms to the Guidelines for Accurate and Transparent Health Estimate Reporting (GATHER) [11].

### Age- and sex-specific prevalence of elevated BP in African adolescents

First, we performed additional subgroup meta-analyses of pre-existing data on the reported prevalence of elevated BP among adolescents aged 10–19 years living in SSA [10]. We then used them to derive more granular estimates of elevated BP in male and female adolescents aged 10–14 and 15–19 years, respectively. These two age groups were chosen based on the American Academy of Paediatrics guidelines [12]. We then performed an odds ratio meta-analysis to determine the sex-specific prevalence of elevated BP in these two age groups. Full details of the original study [10] include a published protocol (PROSPERO: CRD42022297948; **Online Supplementary Document**) [13] and the main results (for adolescents) reported according to the PRISMA guidelines [14]. We divide the data for this study into these two age groups as originally reported from source studies. We observed a linear relationship between age and BP (Figure S1 in the **Online Supplementary Document**). These data informed our decision to project future cardiovascular events.

### Population data

We retrieved global population data by age group and sex for the 49 SSA countries for 2021 (latest year available) from the World Bank database [15]. We extracted country-specific population data for male and female adolescents aged 10–14 and 15–19 years as the denominator for all estimates/projections, which we then aggregated into the four major United Nations Statistics Division SSA regions: Eastern Africa (16 countries/418 million people in 2021), Central Africa (9 countries/190 million), Western Africa (19 countries/505 million), and Southern Africa (5 countries/67 million).

### Country-specific profiling

In the qualitative-based review of the literature, we prospectively identified a range of key socioeconomic and health indicators/factors that would likely influence, either positively or negatively, the number of adolescents developing elevated BP and subsequently experiencing debilitating or fatal cardiovascular events in adulthood. On this basis, where possible, we ranked each of the 49 countries according to their gross domestic product (GDP) per capita [16], current life expectancy [17], rate of rural to urban migration [18], rates of childhood obesity [19], and daily salt intake [20]. On a quantitative basis, we ranked each of these five indicators (from 1 to 49) and identified the highest and lowest countries (indicating the most favourable or worst profile) to assess their likely influence on population-based BP levels relative to the remainder of the region.

### Future cardiovascular events linked to elevated blood pressure

In the absence of African-specific longitudinal cohort studies reporting cardiovascular events linked to varying BP levels/hypertension (noting that this deficit applies to all age groups), we used the next-best available evidence. Specifically, we applied the sex-specific findings of a systematic review and meta-analysis reported by Lou et al. [21], which provided the rate of future cardiovascular events (including coronary and stroke events per 1000 person-years of follow-up) according to varying BP levels among young adults living in multiple countries worldwide. As an important caveat which reflects the paucity of available data, none of the source data were derived from SSA. We then used these data to project the rate of cardiovascular events among African adolescents with persistently elevated BP transitioning to adulthood [10]. We developed a model to calculate the per-year estimates and confidence intervals for the region based on our previous meta-analysis [10] and another published meta-analysis [21] (**Online Supplementary Document**). Specifically, these data suggest that normotensive (systolic BP/diastolic BP<130/85 mm Hg) male and female adolescents aged 18–45 years will experience 1.13 (95% CI = 1.00–1.28) and 1.17 (95% CI = 0.99–1.39) incident cardiovascular events per 1000 person-years of follow-up. These event rates markedly increase to 1.67 (95% CI = 1.42–1.97) and 1.90 (95% CI = 1.54–2.33) per 1000 person-years among male and female adolescents with grade 1 hypertension (systolic BP/diastolic BP 140–159/90–99 mm Hg), respectively. Applying these data (please note that outputs based on high-normal BP or severe grade 2 hypertension are not reported here), we were able to project the excess number of future cardiovascular events linked to elevated BP among those aged 15–19 years over the next 10–25 years.

### Primary and secondary outcomes

For the primary outcome (estimated number of adolescents in SSA with elevated BP), we applied our age- and sex-specific prevalence rates (and the 95% CI for each) derived from our meta-analyses/meta-regression of BP data to the relevant population cohort within each SSA country and composite region. This produced the total number of affected male and female adolescents aged 10–14 and 15–19 years with elevated BP in 2021 in an individual country and on a regional basis. We then applied the expected rate of incident cardiovascular events per 1000 person-years of follow-up on a sex-specific basis derived from the meta-analyses recently reported by Lou et al. [21] to the estimated number of adolescents aged 15–19 years (the cohort that will transition to adulthood within the next 5–10 years) to calculate the excess number of future cardiovascular events within this cohort if their BP remained elevated compared to becoming normotensive (and therefore at less risk of future cardiovascular events). First, we multiplied the at-risk cohort by the base event rate to obtain the non-sex-specific cardiovascular event estimates. We then applied sex-specific risk ratios to these estimates to obtain sex-specific estimates of cardiovascular events. Since the base event rate was 1000 person-years, we divided the estimates by 1000 to obtain annual event estimates and derive 10- and 25-year projections. We rounded up the estimates using a ceiling function to minimise round-off errors by applying the following formula:

*Per-year estimates* = *CEILING.MATH*((*at-risk cohort* × *event rate* × *risk ratio*) / (1000))

We conducted all analyses and constructed all graphical plots using Microsoft Excel, version 2308 (Redmond, Washington, USA).

## RESULTS

The estimated prevalence of elevated BP among those aged 10–14 years and 15–19 years living in SSA increased from 7.1% (95% CI = 4.8–9.7) to 12.7% (95% CI = 10.4–15.3). On a sex-specific basis, the estimated prevalence of elevated BP among male adolescents aged 10–14 and 15–19 years was 7.2% (95 CI = 4.9–9.9) and 13.0% (95% CI = 10.6–15.6), respectively, and 6.9% (95% CI = 4.7–9.5) and 12.5% (95% CI = 10.2–15.0) among female adolescents aged 10–14 and 15–19 years, respectively. As shown in Figure S2 in the **Online Supplementary Document**, consistent with the observed age-related gradient in elevated BP, we estimate that mean systolic BP/diastolic BP levels among SSA adolescents rises from 103 (95% CI = 100–106)/65 (95% CI = 63–67) mmHg to 118 (95% CI = 115–121)/69 (95% CI = 67–71) mmHg among those aged 10–14 and 15–19 years, respectively (see Table S1 in the **Online Supplementary Document** for sex-specific estimates).

Mainland SSA countries with the lowest to highest proportion of adolescents in the total population were South Africa and South Sudan (ranging from a low to a high of 17.2% to 27.6% of their total populations, compared to an SSA average of 23.2%) (**Table 1**). In absolute terms, Nigeria, the Democratic Republic of Congo, and Ethiopia had the highest number of adolescents in 2021 (100 million combined). Except for sodium intake, the island countries of Seychelles and Mauritius had more favourable indicators than continental SSA countries. Accordingly, the Central African Republic, South Sudan, Burundi, and Uganda had multiple unfavourable indicators relative to their counterparts (some of which were offset by factors such as relatively lower levels of urbanisation).

**Table 1:** The population profiles and broad health and socioeconomic indicators for each SSA country*

Country	Total population	Adolescents (10–19 y), n (%)	GDP per capita in USD	Life in years	Urban-dwelling (%)	Annual obesity (%) ↑	Na (g/d)	Key socio-economic indicators for future cardiovascular health and longevity
West Africa								
*Benin*	12 996 895	2 924 949 (22.5)	1319	59.8	50	7.3	2.85	
*Burkina Faso*	22 100 683	5 296 365 (24.0)	893	59.3	32	6.8	2.88	
*Cabo Verde*	587 925	106 516 (18.1)	3293	74.1	68	7.4	3.25	Adolescents ↓, life expectancy ↑, Na intake ↑
*Cote d'Ivoire*	27 478 249	6 552 595 (23.9)	2549	58.6	53	7.0	2.80	
*Gambia*	2 639 916	639 159 (24.2)	772	62.1	64	7.6	3.07	
*Ghana*	32 833 031	7 078 886 (21.6)	2363	63.8	59	7.0	2.35	
*Guinea*	13 531 906	3 145 763 (23.3)	1189	58.9	38	7.4	2.77	
*Guinea-Bissau*	2 060 721	482 783 (23.4)	795	59.7	45	8.0	3.03	
*Liberia*	5 193 416	1 250 379 (24.1)	676	60.8	53	6.1	2.68	Obesity ↓
*Mali*	21 904 983	5 366 358 (24.5)	874	58.9	45	7.8	3.15	Na intake ↑
*Mauritania*	4 614 974	1 105 424 (24.0)	2166	64.4	57	7.1	2.97	
*Niger*	25 252 722	6 071 612 (24.0)	591	61.6	17	8.3	2.92	Urban ↓
*Nigeria*	213 401 323	49 903 610 (23.4)	2066	52.7	54	8.3	2.82	Life expectancy ↓
*Senegal*	16 876 720	3 915 018 (23.2)	1637	67.1	49	6.6	3.15	Life expectancy ↑, Na intake ↑
*Sierra Leone*	8 420 641	1 950 450 (23.2)	480	60.1	44	6.8	2.51	GDP ↓
*Togo*	8 644 829	1 953 770 (22.6)	973	61.6	44	7.7	2.78	
*Total*	418 000 000	97 000 000 (23.4)	1415	60.4	50	7.4	2.87	
Central Africa								
*Angola*	34 503 774	7 989 848 (23.2)	1954	61.6	68	8.5	2.49	Obesity ↑
*Cameroon*	27 198 628	6 271 583 (23.1)	1667	60.3	59	6.9	2.09	
*Central African Republic*	5 457 154	1 453 197 (26.6)	461	53.9	43	7.7	2.80	Adolescents ↑, GDP ↓, life expectancy ↓
*Chad*	17 179 740	4 101 288 (23.9)	686	52.5	24	7.4	2.87	Life expectancy ↓
*Democratic Republic of the Congo*	95 894 118	22 085 713 (23.0)	577	59.2	47	8.1	2.42	
*Republic of the Congo*	5 835 806	1 336 212 (22.9)	2290	63.5	69	8.0	2.25	
*Equatorial Guinea*	1 634 466	331 032 (20.3)	7507	60.6	74	7.9	2.30	GDP ↑, urban ↑
*Gabon*	2 341 179	468 069 (20.0)	8635	65.8	91	6.2	2.01	GDP ↑, urban ↑, obesity ↓
*Sao Tome and Principe*	223 107	53 311 (23.9)	2361	67.6	76	7.4	2.36	Life expectancy ↑, rrban ↑
*Total*	190 000 000	44 000 000 (23.2)	2904	60.6	68	7.7	2.36	
East Africa								
*Burundi*	12 551 213	3 071 563 (24.5)	222	61.7	14	8.4	1.73	GDP ↓, urban ↓, ibesity ↑, Na Intake ↓
*Comoros*	821 625	174 147 (21.2)	1578	63.4	30	7.6	1.67	Na intake ↓
*Djibouti*	1 105 557	225 911 (20.4)	3150	62.3	78	3.9	2.36	Urban ↑, ibesity ↓
*Eritrea*	3 620 312	908 886 (25.1)	644	66.5	43	8.3	2.37	Adolescents ↑
*Ethiopia*	120 283 026	28 113 573 (23.4)	925	65.0	23	7.2	2.27	
*Kenya*	53 005 614	12 725 241 (24.0)	2082	61.4	29	8.2	1.48	Na intake ↓
*Madagascar*	28 915 653	6 582 541 (22.8)	501	64.5	40	8.3	2.20	
*Malawi*	19 889 742	5 043 518 (25.4)	635	62.9	18	8.2	1.66	Adolescents ↑, urban ↓, Na intake ↓
*Mauritius*	1 266 060	170 227 (13.5)	9106	73.7	41	5.9	5.45	Adolescents ↓, GDP ↑, life Expectancy ↑, obesity ↓, Na intake ↑
*Mozambique*	32 077 072	7 583 151 (23.6)	492	59.3	38	7.3	2.24	GDP ↓
*Rwanda*	13 461 888	3 120 083 (23.2)	822	66.1	18	8.4	1.60	Urban ↓, obesity ↑, Na Intake ↓
*Seychelles*	99 258	13 842 (14.0)	14653	73.4	58	4.8	4.34	Adolescents ↓, GDP ↑, life expectancy ↑, obesity ↓, Na intake ↑
*Somalia*	17 065 581	4051 268 (23.7)	447	55.3	47	7.8	2.07	GDP ↓
*South Sudan*	10 748 272	2 858 044 (27.6)	1072	55.0	21	7.9	2.37	Adolescents ↑, life expectancy ↓, urban ↓
*Sudan*	45 657 202	10 078 997 (22.1)	752	65.3	36	7.9	2.37	
*Tanzania*	63 588 334	15 031 014 (23.6)	1099	66.2	37	8.4	2.75	
*Uganda*	45 853 778	11 644 953 (25.4)	884	62.7	26	8.4	2.11	Adolescents ↑, obesity ↑
*Zambia*	19 473 125	4 697 389 (24.1)	1137	61.2	46	6.9	2.27	
*Zimbabwe*	15 993 524	3 788 470 (23.7)	1774	59.3	32	8.1	3.10	
*Total*	505 000 000	119 000 000 (23.7)	2209	62.9	36	7.9	2.27	
Southern Africa								
*Botswana*	2 588 423	511 711 (19.8)	6805	61.1	72	7.7	2.53	Adolescents ↓, urban ↑
*Eswatini*	1 192 271	259 203 (21.7)	3978	57.1	25	8.2	2.53	
*Lesotho*	2 281 454	469 976 (20.6)	1094	53.1	30	8.2	2.62	Life expectancy ↓
*Namibia*	2 530 151	509 476 (20.1)	4866	59.3	54	9.2	2.64	Obesity ↑
*South Africa*	59 392 255	10 222 180 (17.2)	7055	62.3	68	8.2	2.48	Adolescents ↓, GDP ↑
*Total*	67 000 000	11 000 000 (17.6)	4760	59.3	54	8.3	2.53	
Entire region	1 182 000 000	273 000 000 (23.2)	2338	61.8	46	7.5	2.59	

GDP – gross domestic product, Na – sodium, ↑ – the higher probability of elevated blood pressure, ↓ – the lower probability of elevated blood pressure

*The top five and bottom five arrows indicate countries with a higher or lower probability (relative to the average levels applied) of the number of adolescents with elevated BP and its future consequences.

**Figure 2:**
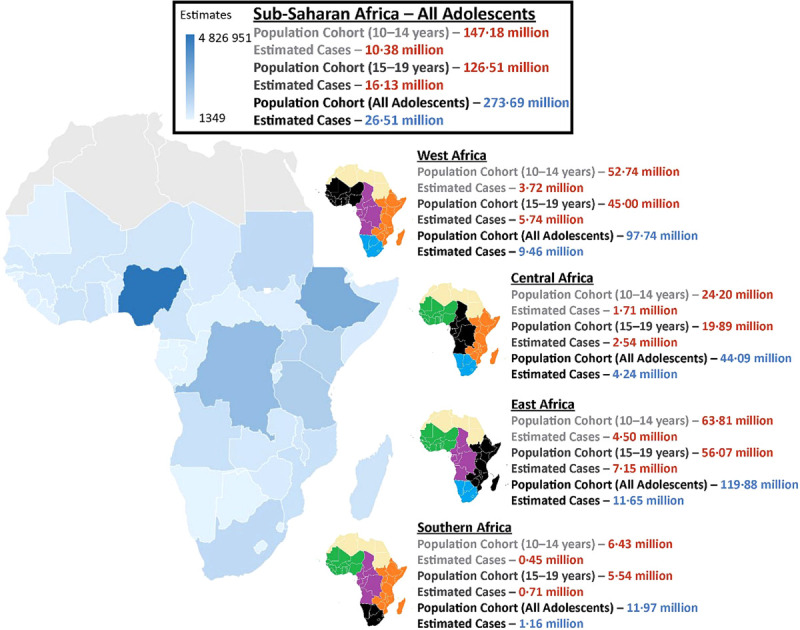
The population profile (including the proportion of adolescents) and estimated number of adolescents of different ages (10–14 years and 15–19 years) and sex groups with elevated BP in each subregion (west, east, central, and south) of SSA.

Overall, an estimated 10.4/147 million (7.1%) adolescents aged 10–14 years and 16.13/127 million (12.8%) adolescents aged 15–19 years in SSA have elevated BP (**Figure 2**). Reflecting the population distribution/dynamics of SSA, the highest number of adolescents (ages 10–19 years) with elevated BP live in East Africa (11.7 million) and West Africa (9.5 million) compared to 2.2 million and 1.2 million cases in Central and Southern Africa, respectively.

**Figure 3:**
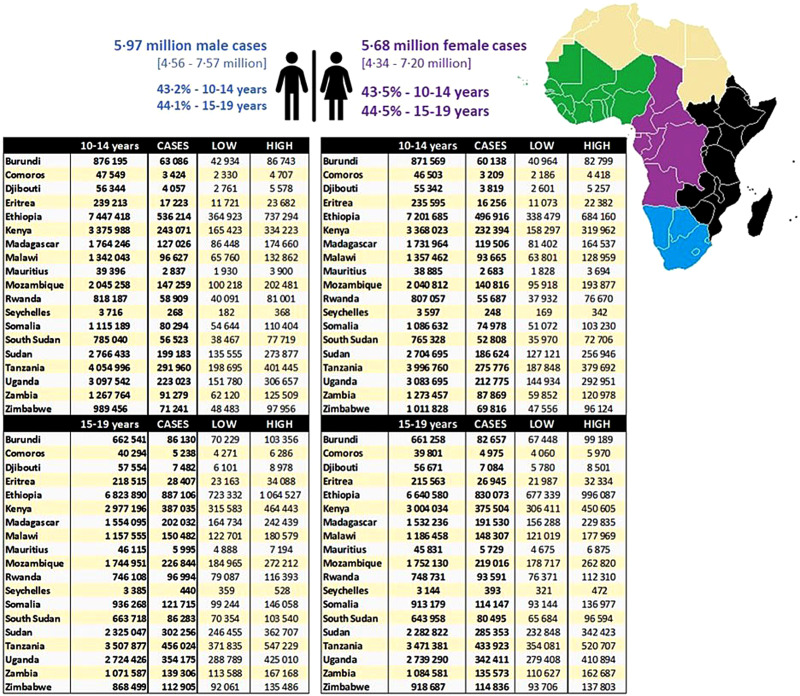
The population distribution of adolescents with elevated blood pressure in East Africa. They were divided into adolescent males and females, and further, into two different age groups (10–14 years and 15–19 years). It also contains the 95% CI.

An estimated 4.9 million male and 4.6 million female adolescents with elevated BP live in the 14 countries comprising West Africa. Together, they represent 35.3–36.1% of all adolescents with elevated BP in SSA (Figure S3 in the **Online Supplementary Document**), with five countries having >200 000 adolescents with elevated BP. In the more sparsely populated Central Africa (Figure S4 in the **Online Supplementary Document**) the more even ratio of 2.2 million male and 2.1 million female adolescents represents 15.6–16.5% of all adolescents with elevated BP; by far, the largest proportion of this population lives in the Democratic Republic of Congo, Cameroon, and Angola. Almost half of the estimated number of adolescents with elevated BP (43.2–44.5%) in SSA live in one of the 19 populous countries of East Africa (**Figure 3**), with a total of almost 5.97 and 5.7 million male and female adolescents, respectively. Ethiopia alone has more than 1 million cases. In contrast, the less populous and relatively wealthier region of Southern Africa (Figure S5 in the **Online Supplementary Document**) has an estimated total of 600 000 and 560 000 male and female adolescents with elevated BP, respectively, with most living in South Africa.

**Figure 4:**
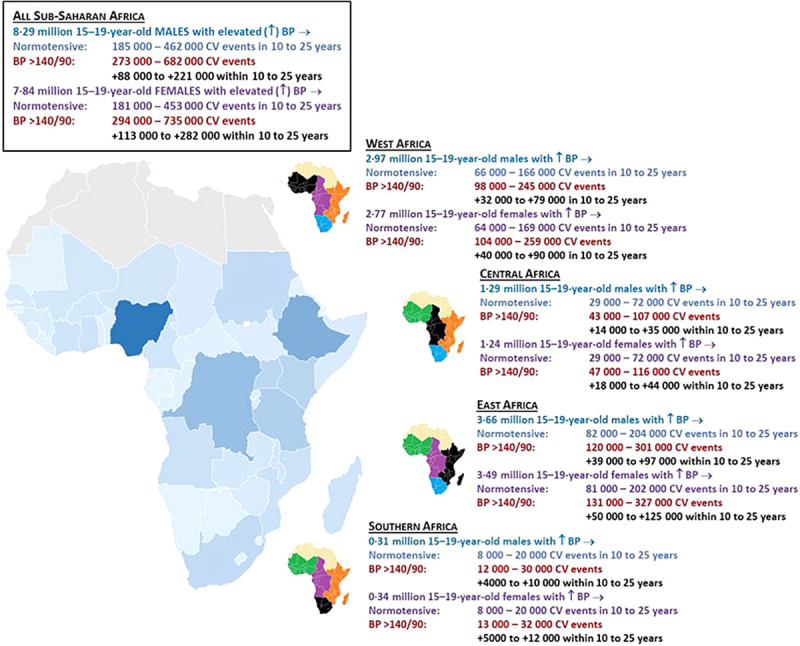
The projected pattern of cardiovascular events within 10 to 25 years among male and female adolescents aged 15–19 years with elevated BP, if they enter adulthood with a favourable/normotensive BP (>120/80 to <139/89 mm Hg) vs unfavourable BP (hypertensive BP>140/90 mm Hg) profile. All figures are rounded to the nearest thousand.

Assuming that the 16.13 million adolescents in SSA aged 15–19 years that are currently estimated to have elevated BP transition into adulthood as normotensive and remain so, our projections indicate that a minimum of 185 000 (95% CI = 123 000–261 000) male and 181 000 (95% CI = 115 000–269 000) female adolescents will experience an incident cardiovascular event (due to other risk factors) within 10 years of reaching (early) adulthood, which may increase to 462 000 (95% CI = 307 000–653 000) and 453 000 (95% CI = 288 000–671 000), respectively, within 25 years. However, if their BP trajectory remains elevated with increasing age (as already indicated by their actual BP levels) and they all progress to grade 1 hypertension, total cardiovascular events among male adolescents are projected to rise from 273 000 (95% CI = 174 000–401 000) to 682 000 (95% CI = 436 000–1.001 million) within the timeframe of 10–25 years. Similarly, the projected number of cardiovascular events among female adolescents will rise from 294 000 (95% CI = 179 000–450 000) to 735 000 (95% CI = 448 000–1.125 million) within 10–25 years. On this basis, among males, we estimate that there will be 88 000 (95% CI = 52 000–141 000) to 221 000 (95% CI = 129 000–352 000) more cardiovascular events within that timeframe if BP levels remain elevated. Among females the equivalent figures are 113 000 (95% CI = 64 000–181 000) to 282 000 (95% CI = 160 000–454 000) more cardiovascular events within that timeframe. The projected sex-specific distribution of excess cardiovascular events attributable to persistently elevated BP within this age cohort across each major region is presented in **Figure 4** and Table S2 in the **Online Supplementary Document** for country-specific data.

## DISCUSSION

Despite the high disease burden linked to hypertension among adults living in SSA [6], there is a paucity of information on the future burden and consequences of elevated BP among adolescents then transitioning to adulthood. To the best of our knowledge, this is the first study to estimate this specific burden of disease and project the likely consequences of more cardiovascular events occurring if these individuals enter adulthood with uncontrolled hypertension. To derive our population estimates, we first performed a more granular analysis of a previously published meta-analysis data [10]. We therefore estimate that 6.9–7.2% and 12.5–13.0% of adolescents aged 10–14 and 15–19 years living in SSA, respectively, have elevated BP, with higher rates observed in males. When applied to population data, this translates to a projected total of 13.6 million and 12.9 million male and female adolescents with elevated BP in SSA. With most adolescents residing in East Africa, several factors including differential rates of economic wealth [16], urbanisation [18], rising obesity levels [19], and excess sodium intake [20] are likely to influence BP levels across SSA. However, our data provide a robust starting point for action to comprehensively map the patterns and likely consequences of elevated BP in young individuals in these largely underprivileged/low-resource regions. Although various methods can be used to generate these estimates, including global disease burden projections [22], our projection method strictly followed those recommended by the GATHER guidelines [11].

By specifically focussing on the 16.13 million adolescents aged 15–19 years with elevated BP approaching adulthood, we estimate that within 25 years (i.e. when they reach the age of 40–44 years), even if their BP normalises, approximately 1 million (with more male than female adolescents affected) will experience a potentially debilitating or fatal cardiovascular event. However, as the observed BP trajectory data supported by external studies [21] suggests, if they collectively develop a BP>140/90 mm Hg, half a million more cardiovascular events would occur in the same timeframe, with many more occurring thereafter. Consistent with the observed disease pattern in SSA [2], this scenario suggests that more female than male adolescents would subsequently experience a cardiovascular event. Notably, although our projections reflect longitudinal observations from a global cohort of 4.5 million young adults [21], no Africa-specific data are currently available to further refine (or challenge) them. Given that our projections are consistent with the enormous burden of premature forms of heart disease [23] and stroke [24] among Africans with a history of undetected/untreated hypertension [9], there remains an urgent need to address the knowledge gaps regarding hypertension and its consequences in SSA.

Some reports suggest that continental Africa is home to some of the highest adult BP levels in the world [25], although a recent meta-analysis found a pooled prevalence of 30% (95% CI = 27–34) hypertension among adults living in SSA [9]. Irrespective of the exact number, SSA (comprising 49 out of 55 of all African countries) has some of the lowest rates of diagnosing, treating, and achieving BP control worldwide [26]. Indeed, despite ambitious development goals, some countries in the region have witnessed little or no improvement in this regard over the past few years [2]. Our findings (based on contemporary BP surveillance studies) corroborate and quantify the magnitude of this problem, both now and in the future. Hypertensive heart disease with consequent heart failure and stroke [6] is a common and serious consequence of elevated BP in relatively young individuals (aged <60 years) in SSA. Among those presenting to the hospital with heart failure, the case fatality is reported to be as high as 18%, with even higher in-hospital (33%) and 28-day (50% overall and 72% for haemorrhagic stroke) case fatalities associated with a cerebrovascular event [27].

If we are to address poor cardiovascular health outcomes in SSA, more cost-effective preventative strategies need to be implemented. Critically, a higher BP detected in early adulthood strongly correlates with an increased risk of all cardiovascular outcomes, such as stroke and heart failure later in life [21].

As shown by a recent systematic review and meta-analysis on the cost of treating uncomplicated hypertension in SSA [28], there is insufficient evidence to definitively determine the true ‘cost’ of treating or not treating hypertension in SSA. The reported cost of antihypertensive treatment ranges from USD 1.70–97.60 and USD 0.09–193.55 per month for patients and providers, respectively [28]. If the suggested average treatment cost of USD 25.00 per month is applied, the cost of treating the estimated 16.13 million adolescents aged 15–19 years with elevated BP approaching adulthood in SSA over 25 years would be enormous at USD 1.21 billion. However, based on a recent South African study [29], the cost of their first, cardiovascular event attributable to hypertension (i.e. without considering any further personal to socio-economic impact including substantive premature mortality, loss of productivity and quality-of-life) would be USD 2.82 billion (range USD 1.62–4.51 billion, based on lowest-to-highest cost/event). Therefore, the cost of doing nothing is substantive, with the cost dynamics (pending formal health-economic analyses) likely to favour primary prevention combined with targeted secondary prevention [30]. Any response to the data provided here will need to consider the broader issue of who will be responsible for identifying adolescents with elevated BP and how they will be managed in the longer term to maintain optimal BP levels that reduce the risk of future cardiovascular events. This will require an increased focus on surveillance and development of a (scalable/widely distributed) health workforce across SSA that addresses the broader issue of cardiovascular risk factors, in addition to national policies (e.g. salt reduction/health lifestyle promotion [20]) that collectively can reduce the overall need for individualised treatment. A range of specific initiatives that already screen adolescents in the region for other important conditions such as rheumatic heart disease can readily incorporate BP monitoring. BP screening could also be routinely conducted in those aged 10–12 years as part of their enrolment before secondary school in the region. Concurrently, initiatives to prompt health workers in SSA to actively screen and optimally manage hypertension in young individuals are urgently required.

When interpreting these data, it is important to note that the original meta-analysis included data from 10 of the 49 SSA countries [10]. Therefore, many SSA countries, mostly low-income/low-resource countries, are under-represented when generating specific data. Moreover, SSA is a highly diverse region, and ethnic and cultural factors are likely to influence the development of high BP. This is important, considering that we conducted further meta-analyses to derive age- and sex-specific estimates in adolescents from an SSA-wide perspective. However, our results are consistent with those of most previous studies conducted in this region. We also relied heavily on World Bank databases [15] for adolescent population data, GDP, and urbanisation data, although not all countries report these key parameters to the World Bank for various reasons [31]. Nevertheless, this database remains one of the largest and most widely used [15]. Although we applied the event rates and risk ratios derived from a global meta-analysis of follow-up studies (according to BP levels) of 4.5 million young adults, none of the source studies/cohorts were derived from SSA cohorts. However, consistent with our findings, surveillance studies in SSA have consistently demonstrated that cardiovascular events linked to hypertension occur at an earlier age, with more women than men affected, compared to other regions of the world [32]. To partially mitigate the lack of definitive data, we applied 95% CIs to generate full-range low-to-high estimates [33].

## CONCLUSIONS

By applying the best available evidence combined with population data, we estimated that, of the approximately 147 million adolescents aged 10–14 years living in SSA in 2021, 10.4 million (7.1%) had elevated BP, which increased to 12.8% (16.1/127 million) among those aged 15–19 years. We further estimated that in the 15–19-year cohort alone, there will be 201 000–503 000 more cardiovascular events in the next 10–25 years if elevated blood pressure/hypertension remains undetected and untreated. To confirm our findings, a wider geographic distribution of methodologically standardised studies must be conducted in this potentially vulnerable and pivotal age group. There is sufficient evidence to prompt urgent efforts to proactively detect and optimally treat elevated blood pressure/hypertension in younger individuals living in SSA.

## Additional material


Online Supplementary Document
