# Supplementary material for: Projected burden and distribution of elevated blood pressure levels and its consequence among adolescents in sub-Saharan Africa
Source: J Glob Health. 2024 Jun 28;14:04136. doi: 10.7189/jogh.14.04136 (PMC11212112; doi:10.7189/jogh.14.04136)
Supplement: Online Supplementary Document [file jogh-14-04136-s001.zip › jogh-14-04136-s001.pdf]

Supplementary Material – Table of Content

1. PROSPERO for previously published meta-analysis

2. Projection model

3. Supplementary Figure S1 – Subgroup meta-analysis on prevalence by age – Page 2

4. Supplementary Figure S2 – Subgroup meta-analysis on blood pressure by age – Page 3

5. Supplementary Table S1 – Sex-specific estimates – Page 4

6. Supplementary Table S2 – Country specific data – Page 5 – 9

7. Supplementary Figure S3 – Population distribution of cases in West Africa – Page 10

8. Supplementary Figure S4 – Population distribution of cases in Central Africa – Page 11

9. Supplementary Figure S5 – Population distribution of cases in Southern Africa – Page 12

PROSPERO for previously published meta-analysis

A Contemporary Analysis of the Geographic Distribution and Characteristics of Elevated Blood Pressure & Hypertensive Heart Disease in Sub-Saharan Africa: A Systematic Review and Meta-Analysis

Simon Stewart, Laura Waite, Alexander Chen, Yih-Kai Chan

Citation

Simon Stewart, Laura Waite, Alexander Chen, Yih-Kai Chan. A Contemporary Analysis of the Geographic Distribution and Characteristics of Elevated Blood Pressure & Hypertensive Heart Disease in Sub-Saharan Africa: A Systematic Review and Meta-Analysis. PROSPERO 2022 CRD42022297948 Available from: https://www.crd.york.ac.uk/prospero/display_record.php?ID=CRD42022297948

Review question

What is the reported geographic distribution and characteristics of elevated blood pressure and associated heart disease among individuals aged ten or more years in sub-Saharan Africa?

Searches

Search criteria:

(1). Primarily focused on hypertension and/or hypertensive heart disease

(2). Research conducted on individuals living in sub-Saharan Africa only

(3). Studies reporting on a minimum of 100 (African) subjects.

(4). Prospective systematic surveillance studies (with or without health outcomes reported). These include surveillance studies, prevalence studies, population attributable risk studies, incidence studies, epidemiological transition studies, clinical surveillance studies, empirical studies and quantitative studies.

Search database: The National Centre for Biotechnology Information

Search dates: Between 1/1/2010 to 31/12/2020 (inclusive)

Restrictions on the search including language and publication period:

(1). Only papers written in English will be included.

(2). Only papers published between 2010 and 2020 (inclusive) will be included.

Whether searches will be re-run prior to the final analysis: Yes

Whether unpublished studies will be sought: No

Types of study to be included

Prospective systematic surveillance studies (with or without health outcomes reported). These include surveillance studies, prevalence studies, population attributable risk studies, incidence studies, epidemiological transition studies, clinical studies, empirical studies and quantitative studies.

Condition or domain being studied

Hypertension and hypertensive heart disease in sub-Saharan Africa

Participants/population

Inclusion:

Adults: Above 19 years old

Adolescents: Between 10 and 19 years old (inclusive)

Exclusion:

Infants/Children: Below 10 years old

Intervention(s), exposure(s)

Individuals with elevated blood pressure (hypertension) or without evidence of hypertensive heart disease living in sub-Saharan Africa

Comparator(s)/control

Sex and age-matched individuals without hypertension and/or hypertensive heart disease living in sub-Saharan Africa

Context

Sub-Saharan Africa is home to a large portion of the poorest billion in the world. Among many health challenges, there is a rising burden of non-communicable disease. A major component of this burden is due to elevated blood pressures levels (hypertension) and consequent hypertensive heart disease. However, any interpretation of this burden must be tempered by the ability of local to regional health systems and researchers to conduct and report on health surveillance data. In the past decade, the annual number of published papers focussing on hypertension and hypertensive disease in Sub-Saharan Africa has effectively doubled. However, per capita, the number of reports is far below high-income countries. Consequently, there is a likely mismatch in the quantity (and quality) of research across the continent.

Within this context, the proposed systematic review and meta-analysis of available data from Sub-Saharan Africa will focus on – a) contemporary data (published in the last decade) and b) the location and target population (from specific clinical cohorts to population surveillance cohorts) from which it was derived. Specifically, based on contemporary surveillance data, the age, gender, occupation, clinical profile, and socioeconomic status of individuals aged ⩾10 years (excluding infants and children) in Sub-Saharan Africa and found to be free from versus living with hypertension and/or hypertensive heart disease will be studied.

The subsequent distribution and characteristics of hypertension and hypertensive heart disease relative to the socio-economic profile of the 46 countries and 4 major regions with Sub-Saharan Africa will be critically examined.

Main outcome(s)

Based on what is known currently, we hypothesize that our analyses will reveal the following –

(1). The reported burden and characteristics of hypertension and hypertensive heart disease in Sub-Saharan Africa is not evenly distributed throughout the region.

(2). Based on contemporary surveillance data, compared with high-income countries in other regions of the world, overall, hypertension and hypertensive heart disease in Sub-Saharan Africa affects substantially more women and younger individuals.

(3). The age and sex specific prevalence of hypertension in Sub-Saharan Africa correlates with the socioeconomic profile of the population being studied.

(4). The subsequent burden and pattern of reported hypertensive heart disease in the region is markedly different from that reported in high-income countries.

Additional outcome(s)

If confirmed, this review/analysis will support efforts to generate more African-specific guidelines for the prevention and management of hypertension; particularly when considering the specific results and translational implications of the multicentre CREOLE Study led by Prof. Ojji. Moreover, by mapping where hypertension-focused research has been generated relative to the population distribution and socio-economic profile of sub-Saharan Africa, this review will generate invaluable data on where more support for health surveillance, clinical programs and public health policies/interventions are needed across the African continent. Key findings will be shared via the Lancet NCDI Poverty Commission network Co-Chaired by Prof. Mocumbi.

Data extraction (selection and coding)

Study selection:

(1). Laura Waite and Alexander Chen will independently screen records for inclusion, and they will be blinded to each other's' decisions.

(2). Professor Simon Stewart will make the final decision for inclusion.

(3). Final selections will be reviewed by Professors Ojji and Mocumbi.

(4). All selections and comments will be stored in an Excel file.

Data extraction:

(1). The number of study subjects (N), type of study, location (by country and region), cohort, clinical profile, and outcome of interest will be extracted.

(2). Alexander Chen will extract data.

(3). Professor Simon Stewart will review Alexander Chen's extraction.

(4). All extract data will be stored in an Excel file.

Risk of bias (quality) assessment

(1). Methods of randomisation, treatment allocation, and blinding will be assessed for each paper at the study level.

(2). The Cochrane risk of bias tool will be used for assessment.

(3). Alexander Chen will assess the risk of bias.

(4). Professor Simon Stewart will review Alexander Chen's assessments.

Strategy for data synthesis

(1). The number of study subjects (N) will be identified for each paper. Only studies with $\geqslant$100 subjects will be included.

(2). Each study will be into group into specific sub-Saharan country of origin and region (West Africa, East Africa, Central Africa, and Southern Africa) – those involving multiple countries being identified separately.

(3). The total number of independent reports and number of individuals studied for each country and collective region will be calculated and compared with the population in each country/region.

(4). The type of study will be identified (e.g., prospective, cross-sectional, and retrospective) and study cohorts grouped (in hierarchal order) as population-based, community, outpatient cohort or hospital cohort.

(5). Where appropriate, specific clinical cohorts will be identified (e.g., HIV and/or post-partum).

(6). The primary outcome of interest is controlled blood pressure versus uncontrolled blood pressure (office and 24-hour ambulatory systolic/diastolic blood pressure of 140/90 or 130/80 mmHg).

(7). The secondary outcome of interest is a clinical diagnosis of hypertensive heart disease with or without evidence of left ventricular hypertrophy and heart failure.

(8). Where appropriate, the prevalence and incidence of hypertension and hypertensive heart disease will be calculated.

(9). The associated rate of cardiovascular (e.g., ischaemic and haemorrhagic stroke) morbidity and mortality and all-cause mortality will be reported.

(10). The meta, metafor, dmetar packages from the statistical software R will be used for meta-analyses.

To avoid publication bias, Funnel plot and the Egger's test will be performed. The p-value threshold of the Egger test for publication bias will be 0.1. χ^2 test on Cochran's Q statistic will be used to evaluate heterogeneity. I^2 values will be used to quantify heterogeneity, which estimates the percentage of total variation across studies due to true between study differences rather than chance. The I^2 threshold for heterogeneity will be 70%.

Analysis of subgroups or subsets

Overall, we will test the hypothesize that both the reported and actual characteristics and distribution of hypertension and hypertensive heart disease, among individuals living in sub-Saharan Africa, is heterogeneous. On this basis will we specifically focus on the following sub-groups:

(1). Individual countries and geographic regions - comprising East Africa includes Sudan, Ethiopia, Tanzania, Kenya and other 9 countries. West Africa includes Nigeria, Ghana, Burkina Faso, Senegal and other 12 countries. Southern Africa includes South Africa, Malawi, Mozambique and other 7 countries. Central Africa includes Cameroon, Congo, Central Africa Republic and other 6 countries. As the number of hypertensive cases is a discrete variable, therefore, the differences will be calculated and compared between each region. Hypothesis testings will be performed to make statistical inferences.

(2). Age groups – comprising adolescents (aged 10-19 years) and adults (aged 20+ years)

(3). Sex – comprising males and females

(4). Community type – comprising urban versus rural communities/location

(5). Clinical cohorts – comprising common communicable (e.g., HIV and tuberculosis) and non-communicable (e.g., diabetes) disease states.

The 95% confidence interval and 95% prediction interval will be reported together with the pooled prevalence estimates (Freeman-Tukey double arcsine transformation using a random-effects meta-analysis model). Incidence will be reported as the number of new cases/1000 person-years follow-up. Comparison of mean blood pressure levels will be performed with Hedge's g calculated to eliminate scale differences. Univariable meta-regression analysis will be applied to identify and quantify sources of heterogeneity including study and participants' characteristics. A two-side $p<0.05$ will be considered statistically significant.

Contact details for further information

Simon Stewart

simon.stewart64@gmail.com

Organisational affiliation of the review

Torrens University Australia

https://www.torrens.edu.au/

Review team members and their organisational affiliations

Professor Simon Stewart. University of Notre Dame Australia

Ms Laura Waite. South Eastern Melbourne Primary Health Network

Mr Alexander Chen. Torrens University Australia

Dr Yih-Kai Chan. ACU

Collaborators

Professor Justin Beilby. Torrens University Australia

Professor Ana Mocumbi. Universidade Eduardo Mondlane

Professor Dike Ojji. University of Abuja

Type and method of review

Epidemiologic, Meta-analysis, Prevention, Prospective meta-analysis (PMA), Systematic review

Anticipated or actual start date

01 September 2021

Anticipated completion date

31 March 2023

Funding sources/sponsors

Not applicable

Grant number(s)

State the funder, grant or award number and the date of award

Not applicable

Conflicts of interest

None known

Language

English

Country

Australia

Stage of review

Review Completed

Subject index terms status

Subject indexing assigned by CRD

Subject index terms

Africa South of the Sahara; Blood Pressure; Heart Diseases; Humans; Hypertension

Date of registration in PROSPERO

13 January 2022

Date of first submission

13 December 2021

Projection Model

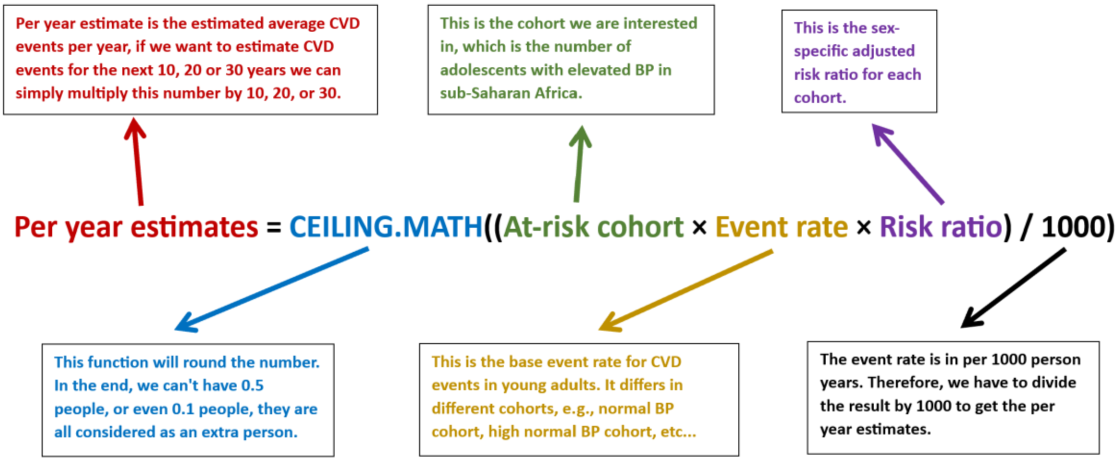

Our projection model uses the at-risk cohort, the event rate, and risk ratio to estimate the average CVD events per year.

Supplementary Figure S1 – Subgroup meta-analysis on prevalence by age

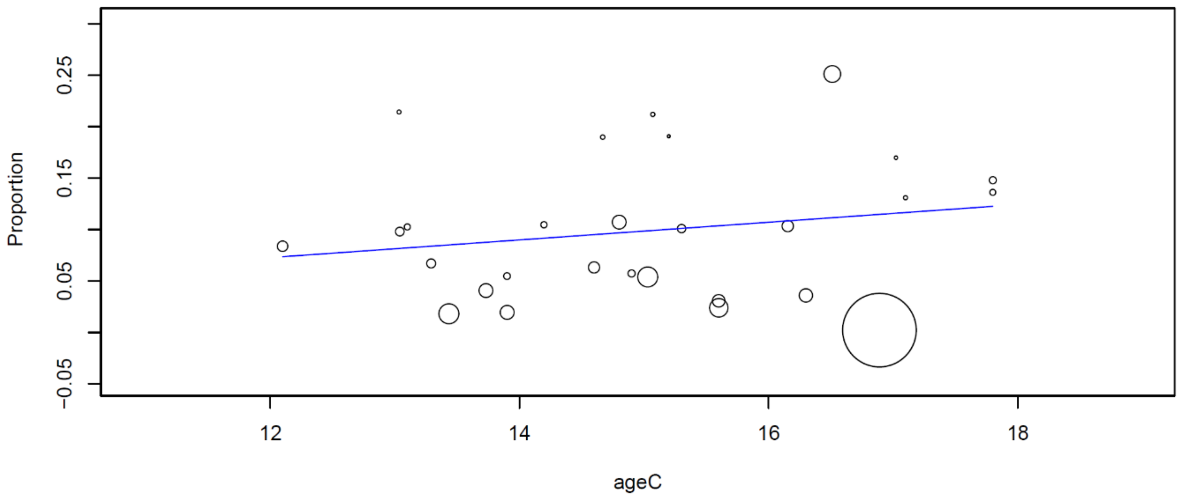

Supplementary Figure S1 demonstrates a linear relationship between age and prevalence among adolescents aged between 10- and 19-years old living in sub-Saharan Africa.

Supplementary Figure S2 – Subgroup meta-analysis on blood pressure by age

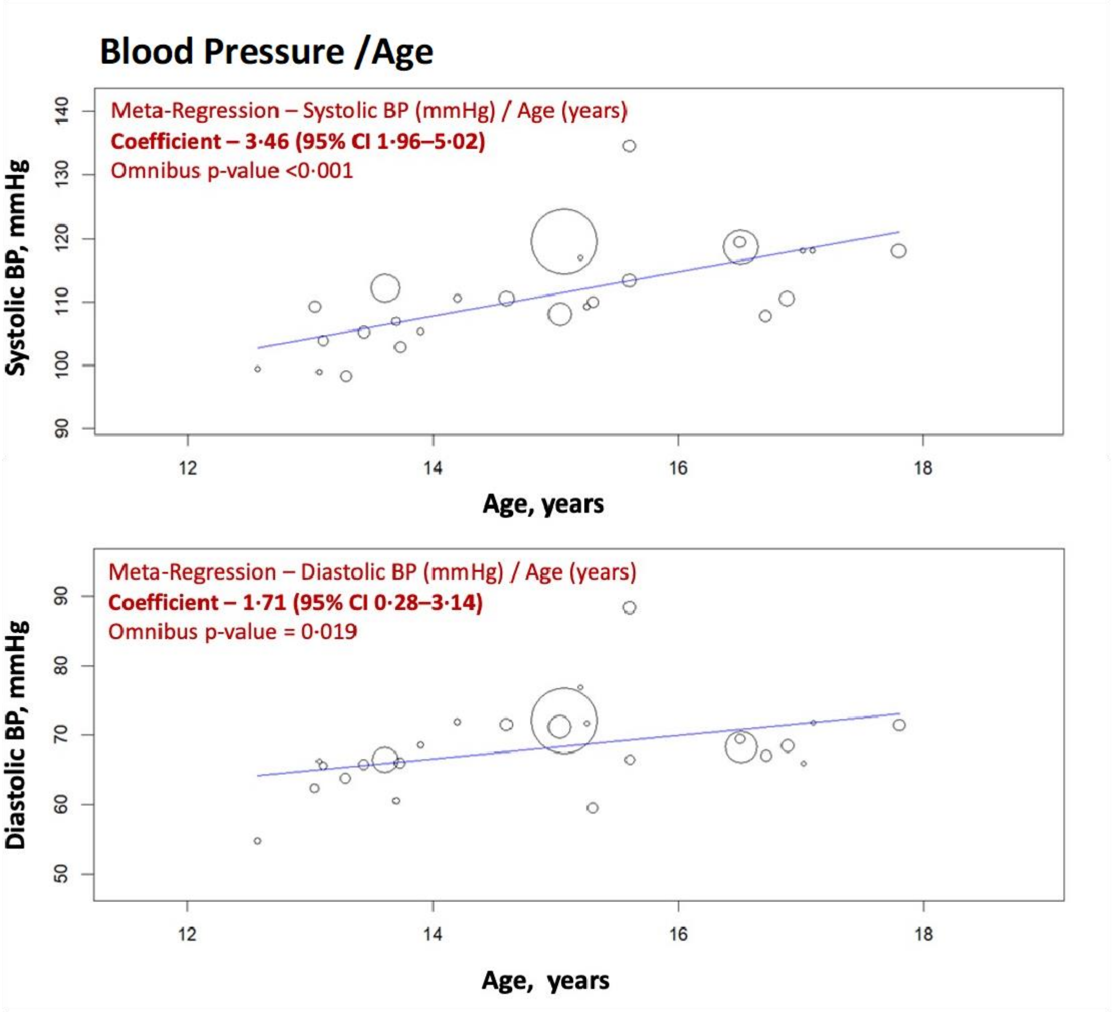

Supplementary Figure S2 demonstrates a linear relationship between age and actual blood pressure levels among adolescents aged between 10- and 19-years old living in sub-Saharan Africa.

Supplementary Table S1 – Sex-specific estimates

	Estimates	Low Estimates	High Estimates
		**Prevalence**	
**Age group 10–14:**			
**Overall**	7.1%	4.8%	9.7%
**Male**	7.2%	4.9%	9.9%
**Female**	6.9%	4.7%	9.5%
**Age group 15–19:**			
**Overall**	12.7%	10.4%	15.3%
**Male**	13.0%	10.6%	15.6%
**Female**	12.5%	10.2%	15.0%
		**Blood pressure levels (SBP/DBP, mmHg)**	
**Age group 10–14:**			
**Overall**	103/65	100/63	106/67
**Male**	104/64	101/62	107/66
**Female**	102/66	99/64	105/68
**Age group 15–19:**			
**Overall**	118/69	115/67	121/71
**Male**	119/68	116/66	122/70
**Female**	117/70	114/68	120/72

Supplementary Table S2 – Country Specific Data

Estimated cases by age groups combined with country and region

10-14 YEARS	AT-RISK COHORT	ALL ESTIMATED CASES	LOW ESTIMATE	HIGH ESTIMATE
Benin	1,567,262	110,524	75,250	152,067
Burkina Faso	2,893,490	204,055	138,930	280,754
Cabo Verde	53,880	3,800	2,587	5,228
Cote d'Ivoire	3,481,787	245,503	167,151	337,783
Gambia	347,353	24,493	16,676	33,700
Ghana	3,733,712	263,284	179,256	362,246
Guinea	1,680,197	118,492	80,675	163,030
Guinea-Bissau	257,197	18,136	12,348	24,953
Liberia	670,271	47,265	32,180	65,032
Mali	2,941,496	207,429	141,227	285,396
Mauritania	602,608	42,492	28,931	58,464
Niger	3,347,094	236,053	160,716	324,779
Nigeria	26,974,485	1,902,414	1,295,251	2,617,477
Senegal	2,109,073	148,726	101,260	204,629
Sierra Leone	1,027,500	72,455	49,331	99,690
Togo	1,051,986	74,181	50,506	102,064
WEST AFRICA	52,739,391	3,719,302	2,532,275	5,117,292
Angola	4,403,905	310,490	211,398	427,198
Cameroon	3,378,708	238,215	162,190	327,757
Central African Rep.	784,827	55,340	37,678	76,141
Chad	2,259,312	159,330	108,479	219,218
Congo (Dem. Rep.)	12,157,541	857,088	583,550	1,179,256
Congo (Rep.)	742,699	52,368	35,655	72,051
Equatorial Guinea	193,616	13,651	9,295	18,784
Gabon	253,370	17,864	12,163	24,579
Sao Tome & Principe	28,224	1,990	1,354	2,737
CENTRAL AFRICA	24,202,202	1,706,336	1,161,762	2,347,721
Burundi	1,747,764	123,224	83,898	169,542
Comoros	94,052	6,633	4,516	9,125
Djibouti	111,686	7,876	5,362	10,835
Eritrea	474,808	33,479	22,794	46,064
Ethiopia	14,649,103	1,033,130	703,402	1,421,454
Kenya	6,744,011	475,465	323,720	654,185
Madagascar	3,496,210	246,532	167,850	339,197
Malawi	2,699,505	190,292	129,561	261,821
Mauritius	78,281	5,520	3,758	7,594
Mozambique	4,086,070	288,075	196,136	396,358
Rwanda	1,625,244	114,596	78,023	157,671
Seychelles	7,313	516	351	710
Somalia	2,201,821	155,272	105,716	213,634
South Sudan	1,550,368	109,331	74,437	150,425

Sudan	5,471,128	385,807	262,676	530,823
Tanzania	8,051,756	567,736	386,543	781,137
Uganda	6,181,237	435,798	296,714	599,608
Zambia	2,541,221	179,148	121,972	246,487
Zimbabwe	2,001,284	141,057	96,039	194,080
EAST AFRICA	63,812,862	4,499,487	3,063,468	6,190,750
Botswana	269,682	19,018	12,948	26,166
Eswatini	136,476	9,622	6,552	13,239
Lesotho	241,288	17,009	11,581	23,402
Namibia	271,468	19,134	13,027	26,327
South Africa	5,510,853	388,660	264,617	534,745
SOUTHERN AFRICA	6,429,767	453,443	308,725	623,879

15-19 YEARS	AT-RISK COHORT	ALL ESTIMATED CASES	LOW ESTIMATE	HIGH ESTIMATE
Benin	1,357,687	173,157	141,240	207,787
Burkina Faso	2,402,875	306,447	249,964	367,737
Cabo Verde	52,636	6,713	5,475	8,055
Cote d'Ivoire	3,070,808	391,646	319,458	469,975
Gambia	291,806	37,213	30,354	44,655
Ghana	3,345,174	426,589	347,961	511,907
Guinea	1,465,566	186,915	152,463	224,297
Guinea-Bissau	225,586	28,766	23,464	34,520
Liberia	580,108	73,977	60,342	88,773
Mali	2,424,862	309,251	252,251	371,102
Mauritania	502,816	64,098	52,283	76,917
Niger	2,724,518	347,493	283,443	416,991
Nigeria	22,929,125	2,924,537	2,385,487	3,509,444
Senegal	1,805,945	230,301	187,853	276,362
Sierra Leone	922,950	117,702	96,007	141,242
Togo	901,784	115,004	93,807	138,005
WEST AFRICA	45,004,246	5,739,809	4,681,852	6,887,769
Angola	3,585,943	457,202	372,933	548,642
Cameroon	2,892,875	368,856	300,871	442,627
Central African Rep.	668,370	85,231	69,522	102,277
Chad	1,841,976	234,909	191,611	281,891
Congo (Dem. Rep.)	9,928,172	1,265,809	1,032,503	1,518,970
Congo (Rep.)	593,513	75,681	61,732	90,817
Equatorial Guinea	137,416	17,531	14,299	21,036
Gabon	214,699	27,376	22,329	32,850
Sao Tome & Principe	25,087	3,197	2,608	3,837
CENTRAL AFRICA	19,888,051	2,535,792	2,068,408	3,042,947
Burundi	1,323,799	168,787	137,677	202,545
Comoros	80,095	10,213	8,331	12,256
Djibouti	114,225	14,566	11,881	17,479
Eritrea	434,078	55,352	45,150	66,422

Ethiopia	13,464,470	1,717,179	1,400,671	2,060,614
Kenya	5,981,230	762,539	621,994	915,048
Madagascar	3,086,331	393,562	321,022	472,274
Malawi	2,344,013	298,789	243,720	358,548
Mauritius	91,946	11,724	9,563	14,069
Mozambique	3,497,081	445,860	363,682	535,032
Rwanda	1,494,839	190,585	155,458	228,703
Seychelles	6,529	833	680	1,000
Somalia	1,849,447	235,862	192,388	283,035
South Sudan	1,307,676	166,778	136,038	200,134
Sudan	4,607,869	587,609	479,303	705,130
Tanzania	6,979,258	889,947	725,916	1,067,936
Uganda	5,463,716	696,586	568,197	835,904
Zambia	2,156,168	274,879	224,215	329,855
Zimbabwe	1,787,186	227,741	185,767	273,289
EAST AFRICA	56,069,956	7,149,391	5,831,653	8,579,273
Botswana	242,029	30,864	25,175	37,037
Eswatini	122,727	15,650	12,765	18,781
Lesotho	228,688	29,154	23,781	34,985
Namibia	238,008	30,338	24,746	36,405
South Africa	4,711,327	600,899	490,142	721,078
SOUTHERN AFRICA	5,542,779	706,905	576,609	848,286

Estimated cases by sex combined with country and region

FEMALES	AT-RISK COHORT	ALL ESTIMATED CASES	LOW ESTIMATE	HIGH ESTIMATE
Benin	1,441,588	136,914	104,530	173,725
Burkina Faso	2,610,658	246,508	187,889	313,200
Cabo Verde	52,589	5,086	3,902	6,427
Cote d'Ivoire	3,240,345	308,248	235,448	390,984
Gambia	316,501	29,932	22,825	38,017
Ghana	3,504,509	334,588	255,832	424,049
Guinea	1,549,196	147,317	112,513	186,874
Guinea-Bissau	239,357	22,788	17,410	28,900
Liberia	618,758	58,788	44,888	74,589
Mali	2,649,183	249,773	190,295	317,456
Mauritania	552,140	52,308	39,907	66,410
Niger	2,984,707	280,927	213,924	357,190
Nigeria	24,499,449	2,320,463	1,770,225	2,946,199
Senegal	1,936,439	183,692	140,196	233,146
Sierra Leone	964,594	92,112	70,435	116,736
Togo	966,127	91,615	69,915	116,289
WEST AFRICA	48,126,140	4,561,059	3,480,134	5,790,191
Angola	3,991,118	375,859	286,261	477,834
Cameroon	3,127,365	296,630	226,385	376,498
Central African Rep.	720,677	68,289	52,103	86,695

Chad	2,023,063	190,528	145,111	242,219
Congo (Dem. Rep.)	11,055,778	1,041,212	793,014	1,323,691
Congo (Rep.)	663,991	62,345	47,442	79,313
Equatorial Guinea	162,985	14,984	11,331	19,155
Gabon	233,354	22,102	16,860	28,061
Sao Tome & Principe	26,904	2,570	1,965	3,257
CENTRAL AFRICA	22,005,235	2,074,519	1,580,472	2,636,723
Burundi	1,532,827	142,795	108,412	181,988
Comoros	86,304	8,184	6,246	10,388
Djibouti	112,013	10,903	8,381	13,758
Eritrea	451,158	43,201	33,060	54,716
Ethiopia	13,842,265	1,326,989	1,015,818	1,680,247
Kenya	6,372,057	607,898	464,708	770,567
Madagascar	3,264,200	311,036	237,690	394,372
Malawi	2,543,920	241,972	184,820	306,928
Mauritius	84,716	8,412	6,503	10,569
Mozambique	3,792,942	359,832	274,635	456,697
Rwanda	1,555,788	149,278	114,303	188,980
Seychelles	6,741	641	490	814
Somalia	1,999,811	189,125	144,216	240,207
South Sudan	1,409,286	133,303	101,654	169,300
Sudan	4,987,517	471,977	359,969	599,369
Tanzania	7,468,141	709,699	541,929	900,399
Uganda	5,822,985	555,186	424,342	703,845
Zambia	2,358,038	223,442	170,479	283,665
Zimbabwe	1,930,515	184,652	141,262	233,927
EAST AFRICA	59,621,224	5,678,525	4,338,917	7,200,736
Botswana	253,113	24,180	18,491	30,641
Eswatini	128,913	12,301	9,404	15,592
Lesotho	236,545	22,770	17,451	28,805
Namibia	257,782	24,540	18,748	31,122
South Africa	5,022,120	476,157	363,355	604,416
SOUTHERN AFRICA	5,898,473	559,948	427,449	710,576

MALES	AT-RISK COHORT	ALL ESTIMATED CASES	LOW ESTIMATE	HIGH ESTIMATE
Benin	1,483,361	146,767	111,960	186,129
Burkina Faso	2,685,707	263,994	201,005	335,291
Cabo Verde	53,927	5,427	4,160	6,856
Cote d'Ivoire	3,312,250	328,901	251,161	416,774
Gambia	322,658	31,774	24,205	40,338
Ghana	3,574,377	355,285	271,385	450,104
Guinea	1,596,567	158,090	120,625	200,453
Guinea-Bissau	243,426	24,114	18,402	30,573
Liberia	631,621	62,454	47,634	79,216
Mali	2,717,175	266,907	203,183	339,042
Mauritania	553,284	54,282	41,307	68,971

Niger	3,086,905	302,619	230,235	384,580
Nigeria	25,404,161	2,506,488	1,910,513	3,180,722
Senegal	1,978,579	195,335	148,917	247,845
Sierra Leone	985,856	98,045	74,903	124,196
Togo	987,643	97,570	74,398	123,780
WEST AFRICA	49,617,497	4,898,052	3,733,993	6,214,870
Angola	3,998,730	391,833	298,070	498,006
Cameroon	3,144,218	310,441	236,676	393,886
Central African Rep.	732,520	72,282	55,097	91,723
Chad	2,078,225	203,711	154,979	258,890
Congo (Dem. Rep.)	11,029,935	1,081,685	823,039	1,374,535
Congo (Rep.)	672,221	65,704	49,945	83,555
Equatorial Guinea	168,047	16,198	12,263	20,665
Gabon	234,715	23,138	17,632	29,368
Sao Tome & Principe	26,407	2,617	1,997	3,317
CENTRAL AFRICA	22,085,018	2,167,609	1,649,698	2,753,945
Burundi	1,538,736	149,216	113,163	190,099
Comoros	87,843	8,662	6,601	10,993
Djibouti	113,898	11,539	8,862	14,556
Eritrea	457,728	45,630	34,884	57,770
Ethiopia	14,271,308	1,423,320	1,088,255	1,801,821
Kenya	6,353,184	630,106	481,006	798,666
Madagascar	3,318,341	329,058	251,182	417,099
Malawi	2,499,598	247,109	188,461	313,441
Mauritius	85,511	8,832	6,818	11,094
Mozambique	3,790,209	374,103	285,183	474,693
Rwanda	1,564,295	155,903	119,178	197,394
Seychelles	7,101	708	541	896
Somalia	2,051,457	202,009	153,888	256,462
South Sudan	1,448,758	142,806	108,821	181,259
Sudan	5,091,480	501,439	382,010	636,584
Tanzania	7,562,873	747,984	570,530	948,674
Uganda	5,821,968	577,198	440,569	731,667
Zambia	2,339,351	230,585	175,708	292,677
Zimbabwe	1,857,955	184,146	140,544	233,442
EAST AFRICA	60,261,594	5,970,353	4,556,204	7,569,287
Botswana	258,598	25,702	19,632	32,562
Eswatini	130,290	12,971	9,913	16,428
Lesotho	233,431	23,393	17,911	29,582
Namibia	251,694	24,932	19,025	31,610
South Africa	5,200,060	513,402	391,404	651,407
SOUTHERN AFRICA	6,074,073	600,400	457,885	761,589

Supplementary Figure S3 – Population Distribution of Cases in West Africa

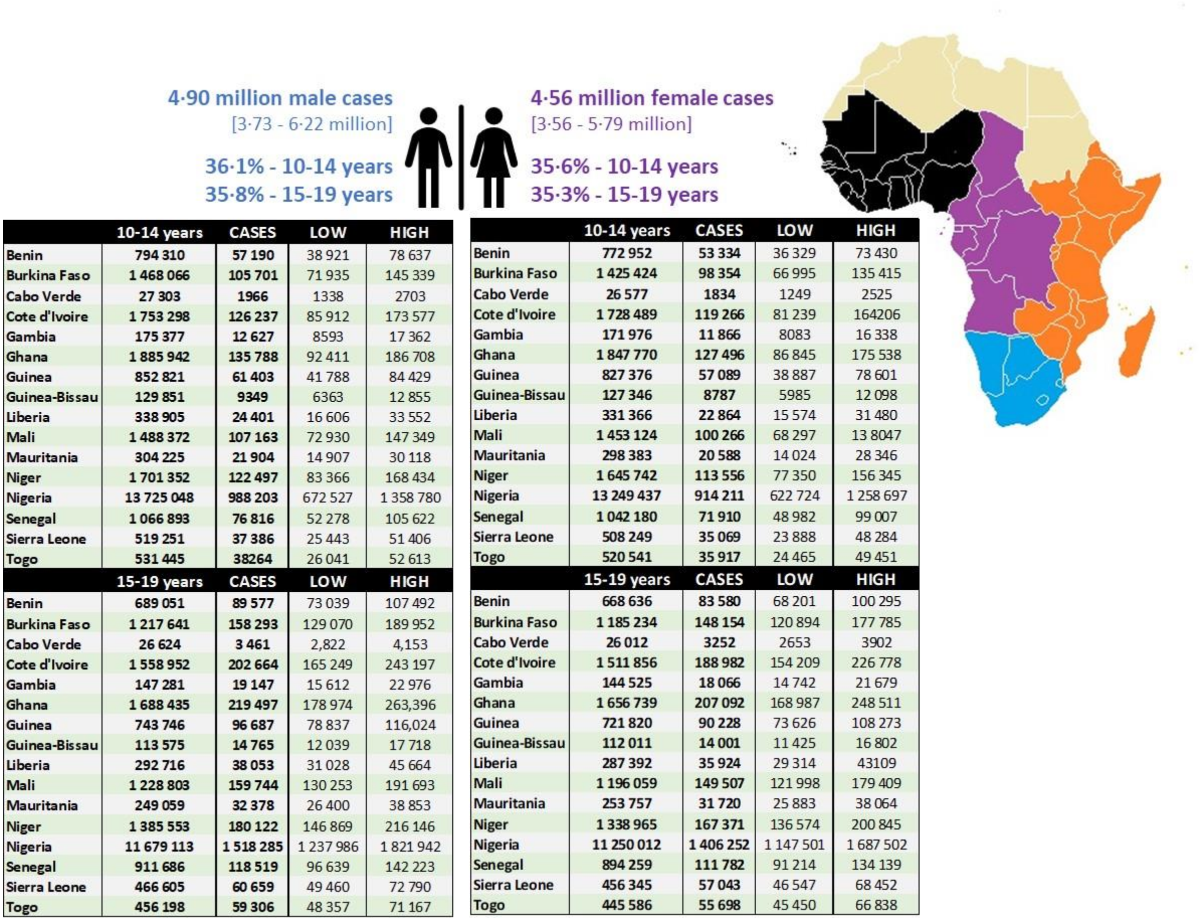

4·90 million male cases
[3·73 - 6·22 million]

36·1% - 10-14 years
35·8% - 15-19 years

4·56 million female cases
[3·56 - 5·79 million]

35·6% - 10-14 years
35·3% - 15-19 years

	10-14 years	CASES	LOW	HIGH
Benin	794 310	57 190	38 921	78 637
Burkina Faso	1 468 066	105 701	71 935	145 339
Cabo Verde	27 303	1966	1338	2703
Cote d'Ivoire	1 753 298	126 237	85 912	173 577
Gambia	175 377	12 627	8593	17 362
Ghana	1 885 942	135 788	92 411	186 708
Guinea	852 821	61 403	41 788	84 429
Guinea-Bissau	129 851	9349	6363	12 855
Liberia	338 905	24 401	16 606	33 552
Mali	1 488 372	107 163	72 930	147 349
Mauritania	304 225	21 904	14 907	30 118
Niger	1 701 352	122 497	83 366	168 434
Nigeria	13 725 048	988 203	672 527	1 358 780
Senegal	1 066 893	76 816	52 278	105 622
Sierra Leone	519 251	37 386	25 443	51 406
Togo	531 445	38264	26 041	52 613
	**15-19 years**	**CASES**	**LOW**	**HIGH**
Benin	689 051	89 577	73 039	107 492
Burkina Faso	1 217 641	158 293	129 070	189 952
Cabo Verde	26 624	3 461	2,822	4,153
Cote d'Ivoire	1 558 952	202 664	165 249	243 197
Gambia	147 281	19 147	15 612	22 976
Ghana	1 688 435	219 497	178 974	263,396
Guinea	743 746	96 687	78 837	116,024
Guinea-Bissau	113 575	14 765	12 039	17 718
Liberia	292 716	38 053	31 028	45 664
Mali	1 228 803	159 744	130 253	191 693
Mauritania	249 059	32 378	26 400	38 853
Niger	1 385 553	180 122	146 869	216 146
Nigeria	11 679 113	1 518 285	1 237 986	1 821 942
Senegal	911 686	118 519	96 639	142 223
Sierra Leone	466 605	60 659	49 460	72 790
Togo	456 198	59 306	48 357	71 167

	10-14 years	CASES	LOW	HIGH
Benin	772 952	53 334	36 329	73 430
Burkina Faso	1 425 424	98 354	66 995	135 415
Cabo Verde	26 577	1834	1249	2525
Cote d'Ivoire	1 728 489	119 266	81 239	164206
Gambia	171 976	11 866	8083	16 338
Ghana	1 847 770	127 496	86 845	175 538
Guinea	827 376	57 089	38 887	78 601
Guinea-Bissau	127 346	8787	5985	12 098
Liberia	331 366	22 864	15 574	31 480
Mali	1 453 124	100 266	68 297	13 8047
Mauritania	298 383	20 588	14 024	28 346
Niger	1 645 742	113 556	77 350	156 345
Nigeria	13 249 437	914 211	622 724	1 258 697
Senegal	1 042 180	71 910	48 982	99 007
Sierra Leone	508 249	35 069	23 888	48 284
Togo	520 541	35 917	24 465	49 451
	**15-19 years**	**CASES**	**LOW**	**HIGH**
Benin	668 636	83 580	68 201	100 295
Burkina Faso	1 185 234	148 154	120 894	177 785
Cabo Verde	26 012	3252	2653	3902
Cote d'Ivoire	1 511 856	188 982	154 209	226 778
Gambia	144 525	18 066	14 742	21 679
Ghana	1 656 739	207 092	168 987	248 511
Guinea	721 820	90 228	73 626	108 273
Guinea-Bissau	112 011	14 001	11 425	16 802
Liberia	287 392	35 924	29 314	43109
Mali	1 196 059	149 507	121 998	179 409
Mauritania	253 757	31 720	25 883	38 064
Niger	1 338 965	167 371	136 574	200 845
Nigeria	11 250 012	1 406 252	1 147 501	1 687 502
Senegal	894 259	111 782	91 214	134 139
Sierra Leone	456 345	57 043	46 547	68 452
Togo	445 586	55 698	45 450	66 838

Supplementary Figure S3 shows the population distribution of cases in West Africa. It is divided into males and females and two different age groups (between 10-–14 years and between 15-–19 years).

Supplementary Figure S4 – Population Distribution of Cases in Central Africa

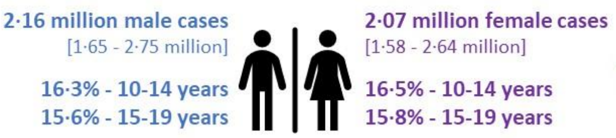

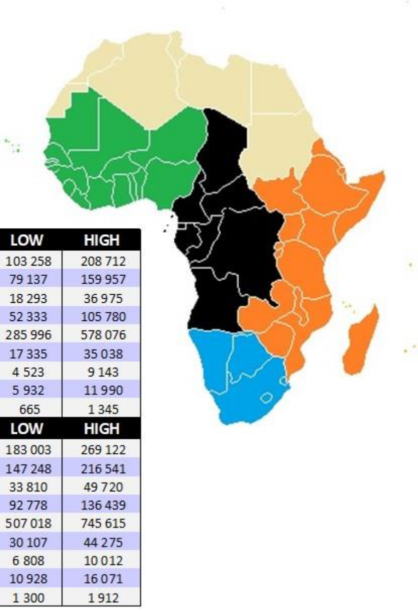

	10-14 years	CASES	LOW	HIGH
Angola	2 206 933	158 899	108 140	218 486
Cameroon	1 694 949	122 036	83 053	167 800
Central African Rep.	395 619	28 485	19 385	39 166
Chad	1 145 842	82 501	56 146	113 438
Congo (Dem. Rep.)	6 072 528	437 222	297 554	601 180
Congo (Rep.)	373 873	26 919	18 320	37 013
Equatorial Guinea	97 379	7 011	4 772	9 641
Gabon	127 157	9 155	6 231	12 589
Sao Tome & Principe	14 065	1 013	689	1 392
	15-19 years	CASES	LOW	HIGH
Angola	1 791 797	232 934	189 930	279 520
Cameroon	1 449 269	188 405	153 623	226 086
Central African Rep.	336 901	43 797	35 712	52 557
Chad	932 383	121 210	98 833	145 452
Congo (Dem. Rep.)	4 957 407	644 463	525 485	773 355
Congo (Rep.)	298 348	38 785	31 625	46 542
Equatorial Guinea	70 668	9 187	7 491	11 024
Gabon	107 558	13 983	11 401	16 779
Sao Tome & Principe	12 342	1 604	1 308	1 925

	10-14 years	CASES	LOW	HIGH
Angola	2 196 972	151 591	103 258	208 712
Cameroon	1 683 759	116 179	79 137	159 957
Central African Rep.	389 208	26 855	18 293	36 975
Chad	1 113 470	76 829	52 333	105 780
Congo (Dem. Rep.)	6 085 013	419 866	285 996	578 076
Congo (Rep.)	368 826	25 449	17 335	35 038
Equatorial Guinea	96 237	6 640	4 523	9 143
Gabon	126 213	8 709	5 932	11 990
Sao Tome & Principe	14 159	977	665	1 345
	15-19 years	CASES	LOW	HIGH
Angola	1 794 146	224 268	183 003	269 122
Cameroon	1 443 606	180 451	147 248	216 541
Central African Rep.	331 469	41 434	33 810	49 720
Chad	909 593	113 699	92 778	136 439
Congo (Dem. Rep.)	4 970 765	621 346	507 018	745 615
Congo (Rep.)	295 165	36 896	30 107	44 275
Equatorial Guinea	66 748	8 344	6 808	10 012
Gabon	107 141	13 393	10 928	16 071
Sao Tome & Principe	12 745	1 593	1 300	1 912

Supplementary Figure S4 shows the population distribution of cases in Central Africa. It is divided into males and females and two different age groups (between 10-–14 years and between 15-–19 years).

Supplementary Figure S5 – Population Distribution of Cases in Southern Africa

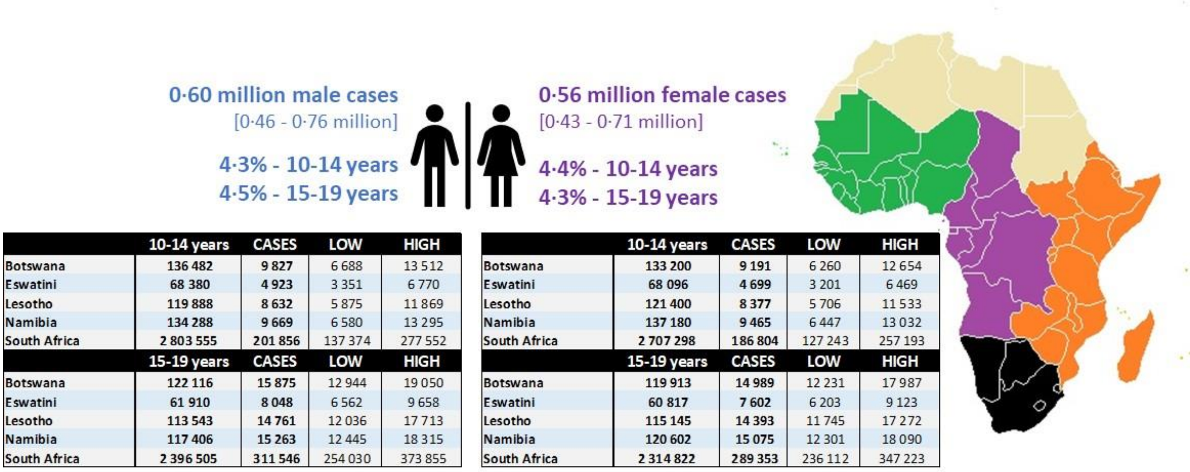

	10-14 years	CASES	LOW	HIGH
Botswana	136 482	9 827	6 688	13 512
Eswatini	68 380	4 923	3 351	6 770
Lesotho	119 888	8 632	5 875	11 869
Namibia	134 288	9 669	6 580	13 295
South Africa	2 803 555	201 856	137 374	277 552
	**15-19 years**	**CASES**	**LOW**	**HIGH**
Botswana	122 116	15 875	12 944	19 050
Eswatini	61 910	8 048	6 562	9 658
Lesotho	113 543	14 761	12 036	17 713
Namibia	117 406	15 263	12 445	18 315
South Africa	2 396 505	311 546	254 030	373 855

	10-14 years	CASES	LOW	HIGH
Botswana	133 200	9 191	6 260	12 654
Eswatini	68 096	4 699	3 201	6 469
Lesotho	121 400	8 377	5 706	11 533
Namibia	137 180	9 465	6 447	13 032
South Africa	2 707 298	186 804	127 243	257 193
	**15-19 years**	**CASES**	**LOW**	**HIGH**
Botswana	119 913	14 989	12 231	17 987
Eswatini	60 817	7 602	6 203	9 123
Lesotho	115 145	14 393	11 745	17 272
Namibia	120 602	15 075	12 301	18 090
South Africa	2 314 822	289 353	236 112	347 223

Supplementary Figure S5 shows the population distribution of cases in Southern Africa. It is divided into males and females and two different age groups (between 10—14 years and between 15-—19 years).
