# Supplementary material for: Projected burden and distribution of elevated blood pressure levels and its consequence among adolescents in sub-Saharan Africa
Source: J Glob Health. 2024 Jun 28;14:04136. doi: 10.7189/jogh.14.04136 (PMC11212112; doi:10.7189/jogh.14.04136)
Supplement: Online Supplementary Document [file jogh-14-04136-s001.zip › jogh-14-04136-s002.pdf]

GATHER Checklist

Objectives and funding

1. Define the indicator(s), populations (including age, sex, and geographic entities), and period(s) for which estimates were made. ✓

2. List the funding sources for the work. ✓

Data inputs

For all data inputs from multiple sources that are synthesised as part of the study:

3. Describe how the data were identified and how the data were accessed. ✓

4. Specify the inclusion and exclusion criteria. Identify all ad-hoc exclusions. ✓

5. Provide information about all included data sources and their main characteristics. For each data source used, report reference information or contact name/institution, population represented, data collection method, year(s) of data collection, sex and age range, diagnostic criteria or measurement method, and sample size, as relevant. ✓

6. Identify and describe any categories of input data that have potentially important biases (eg, based on characteristics listed in item 5). ✓

For data inputs that contribute to the analysis but were not synthesised as part of the study:

7. Describe and give sources for any other data inputs. ✓

For all data inputs:

8. Provide all data inputs in a file format from which data can be efficiently extracted (e.g., a spreadsheet rather than a PDF), including all relevant meta-data listed in item 5. For any data inputs that cannot be shared because of ethical or legal reasons, such as third-party ownership, provide a contact name or the name of the institution that retains the right to the data. ✓

Data analysis

9. Provide a conceptual overview of the data analysis method. A diagram may be helpful. ✓

10. Provide a detailed description of all steps of the analysis, including mathematical formulae. This description should cover, as relevant, data cleaning, data pre-processing, data adjustments and weighting of data sources, and mathematical or statistical model(s). ✓

11. Describe how candidate models were evaluated and how the final model(s) were selected. ✓

12. Provide the results of an evaluation of model performance, if done, as well as the results of any relevant sensitivity analysis. ✓

13. Describe methods of calculating uncertainty of the estimates. State which sources of uncertainty were, and were not, accounted for in the uncertainty analysis. ✓

14. State how analytical or statistical source code used to generate estimates can be accessed. ✓

Results and discussion

15. Provide published estimates in a fi le format from which data can be efficiently extracted. ✓

16. Report a quantitative measure of the uncertainty of the estimates (e.g., uncertainty intervals). ✓

17. Interpret results in light of existing evidence. If updating a previous set of estimates, describe the reasons for changes in estimates. ✓

18. Discuss limitations of the estimates. Include a discussion of any modelling assumptions or data limitations that affect interpretation of the estimates. ✓
